# Touch-flavor transference: Assessing the effect of packaging weight on gustatory evaluations, desire for food and beverages, and willingness to pay

**DOI:** 10.1371/journal.pone.0186121

**Published:** 2017-10-11

**Authors:** Kristina Kampfer, Alexander Leischnig, Björn Sven Ivens, Charles Spence

**Affiliations:** 1 Department of Marketing, University of Bamberg, Feldkirchenstr, 21, Bamberg, Germany; 2 School of Business and Management, Queen Mary University of London, London, United Kingdom; 3 Department of Experimental Psychology, University of Oxford, Oxford, United Kingdom; University of Kansas Medical Center, UNITED STATES

## Abstract

Product packaging serves a number of distinct functions and influences the way in which consumers respond to various product offerings. The research reported here examines whether the haptic characteristics of a non-diagnostic product packaging cue, namely its weight, affects the response of consumers. This article reviews existing research on haptic transference and proposes a conceptual framework to explore how the weight of product packaging affects the flavor of the food or beverages, and, in turn, consumers’ desire for consumption and willingness to pay. Two studies demonstrate that an increase in packaging weight affects both desire and willingness to pay for the product. These effects are serially mediated by perceived flavor intensity and overall flavor evaluation. Based on these insights, implications for the design of food and beverages packaging are discussed.

## Introduction

In recent years, sensory marketing, defined as *“marketing that engages the consumers’ senses and affects their perception*, *judgment and behavior”* ([1], p. 332), has emerged as an important field of research [2]. Research in sensory marketing underscores the notion that subconscious triggers differ in their effects on consumers from more conventional triggers (e.g., [3, 4]). With sensory information being ambiguous (uncertain), and thus open to different interpretations, researchers have also shown that non-diagnostic sensory cues often influence cognitive appraisals [5]. The diagnosticity of a stimulus describes the probability that its use will lead to successful task resolution [6].

This article focuses on product packaging. Specifically, this research develops and tests a conceptual framework that describes the causal chain leading from the weight of product packaging via perceived flavor and flavor evaluation to the desire for food and beverages, and, ultimately, consumers’ willingness to pay for them. Product packaging can be thought of as a combination of various elements blended into a holistic design to achieve a particular sensory effect or outcome [7]. The design of appealing multisensory product packaging builds upon multiple dimensions, such as visual appearance, haptics, and even packaging sounds (e.g., [7–10]). Previous research on the effect of product packaging on consumer responses has focused primarily on visual stimuli (e.g., [11]). For example, research shows that the position of an image of a product on the packaging affects consumers’ perceptions of the visual weight of the product and evaluations of the package [12, 13].

In addition to whatever visual cues may be available, consumers’ responses to haptic product interactions have emerged as an important field of study over the last few years (e.g., [14–16]). Haptic perception plays an important role in product evaluation and can affect consumer behavior by providing information that cannot necessarily be assessed reliably via the consumer’s other senses (e.g., temperature, weight, compressibility; [3, 17]). This research examines how the processing of the non-diagnostic haptic properties of product packaging influences people’s product evaluations and reactions. Specifically, we explore how the weight of food and beverage packaging can influence flavor evaluations and, in turn, consumers’ desire for food and beverages and their willingness to pay for them.

The existing body of research on multisensory product perception demonstrates that the characteristics of product packaging can bias consumers’ flavor evaluations and that weight plays a critical role here [18]. However, existing studies have failed to address the response of consumers to such interventions. What is more, as yet, the extant research provides little insight into the causal processes that underlie such cross-modal effects. Thus, the key questions become if, and how, the weight of the packaging affects the consumer’s response to weight? To answer these questions, the present research reports two experimental studies designed specifically to examine whether an increase in packaging weight would influence desire for food and beverages and willingness to pay. In addition, this research investigates haptic-gustatory sensation transference effects by demonstrating a serial mediating mechanism of flavor intensity and flavor evaluation. Consumers thus have a higher desire and a higher willingness to pay for food and beverages consumed from heavy packaging as they perceive it as more intense and, in turn, evaluate it more favorably.

The contributions of this research to the literature are threefold. First, a review of the literature on haptic transference provides a systematic overview of haptic dimensions and their impact on consumers’ responses. Second, this research adds to the literature on sensation transference by examining the effects of haptic cues on the desire to consume and on willingness to pay. The findings reveal that the weight of product packaging indirectly increases both consumers’ desire for consumption and their willingness to pay through stronger perceived flavor intensity and more favorable overall flavor evaluations. Thus, this research demonstrates that haptic-gustatory sensation transference is a critical mechanism that helps in understanding the causal effects of packaging weight on consumers’ consumption-related and payment-related responses. Third, the two experimental studies reported here add to the existing research on the topic by demonstrating these effects specifically for food and beverages. Prior studies have most commonly focused on food vs. non-food products in general. This research distinguishes between food and beverages, that is, two product categories whose particular characteristics necessitate different modes of consumption.

The remainder of this article is organized as follows. The following section explains the theoretical foundation of this research. Then, attention turns toward the literature on sensation transference and a review of existing empirical work on haptic transference. Next, this study formulates hypotheses describing how packaging weight affects consumers’ perception of flavor intensity, flavor evaluation, and subsequently their desire for food and beverages and their willingness to pay. These hypotheses are tested via two experimental studies. Based on the results, theoretical contributions, managerial implications, and avenues for further research are discussed.

## Theoretical background and hypotheses

### Sensation transference

Sensation transference is a psychological mechanism through which a consumer’s perceptions of one attribute of a stimulus can carry over to influence the same consumer’s perception and rating of another seemingly unrelated attribute of the same stimulus [19]. Sensation transference can occur within a given sensory modality as well as between modalities, and has been shown to influence consumers’ multisensory product experience under various conditions [19, 20]. For example, prior work has established that visual cues, such as rounded design elements, can affect consumers’ product perception [19, 21]. In addition, prior studies relate shapes to tastes and odors. Rounded, voluminous and regular shapes (e.g., circles) are associated with sweetness [22]. Similarly, there is a cross-modal influence of shapes and the semantic hedonic meaning of words that affects perceived sweet taste [23].

Sensory perception is intrinsically affective. The hedonic attributes of a product perceived via one sense (e.g., touch) can affect consumers’ appraisal of a multisensory product experienced by their various senses. This effect, known as “affective ventriloquism”, represents a specific (i.e., hedonic) form of sensation transference [3]. Ventriloquism has been originally used to describe the “dominance” of vision over auditory locations (which, for example, can be observed when a ventriloquist’s voice is mislocalized toward the lip-movements of the ventriloquist’s dummy) and it has been extended to the tactile domain to explain why changing the feel of products or product packaging (through modulation of haptic cues) affect overall multisensory product evaluations [4].

One can sense haptic cues by means of active touch (i.e., when a product is actively explored by an individual), which is the focus of this research, or by passive touch (i.e., when a product or an object is placed on the surface of the skin [24]). Haptic cues that can be directly ascertained by the sense of touch encompass hardness, temperature, texture, and weight [25]. Table 1 provides a summary of research on haptic transference and organizes studies based on the haptic cues examined.

**Table 1:** Overview of studies on haptic transference.

Author(s)	Focus	Empirical basis	Key findings
Lederman & Klatzky (1987) [25]	Haptic exploration	• Study 1: n = 18, three-dimensional stimuli • Study 2: n = 48, three-dimensional stimuli	Hand movements serve as a device for learning about the underlying representation of objects in memory and the processes by which such representations are derived and utilized.
Krishna & Morrin (2008) [14]	Hardness	• Study 1: n = 180, cups with water • Study 2: n = 271, cups with water • Study 3: n = 263, scenario on bottled water • Study 4: n = 225, cups with water	The hardness of the container positively affects product evaluation. This effect is dependent on need for touch and is seen only in individuals with a low need for touch.
Barnett-Cowan (2010) [25]	Hardness	• Study: n = 10, pretzels	The haptic information available when handling food has a role in modulating our perception of it. A fresh pretzel was perceived as staler and softer when the end of the pretzel held in the hand felt stale and soft.
Williams & Bargh (2008) [28]	Temperature	• Study 1: n = 41, cups • Study 2: n = 53, therapeutic pads	Holding a cup of hot (versus iced) coffee results in people being judged as having a “warm” personality. Holding a hot (versus cold) therapeutic pad positively affects the choice of a prosocial gift over a personal reward.
McDaniel & Baker (1977) [27]	Texture	• Study 1: n = 400, potato chips packages • Study 2: n = 100, potato chips packages	The texture of the packaging in which potato chips are presented affects the perception of crispiness and tastiness. Chips in harder-to-open packages are evaluated as fresher and crispier than those in easier-to-open packages.
Zampini, Mawhinney, & Spence (2006) [32]	Texture	• Study: n = 20, deodorant sticks	The roughness of a tool’s handle affects the perceived roughness of its cap. Rougher tool caps rated as significantly less rough when participant holds a tool with a rough (versus smooth) handle.
Tu, Yang, & Ma (2015) [33]	Texture	• Study 1: n = 22, liquid food containers • Study 2: n = 24, liquid food containers	The material of a beverage container affects perceived taste. Dimensions of sweetness are rated higher when a product is presented in a glass container rather than in a paper container.
Biggs, Juravle, & Spence (2016) [13]	Texture	• Study 1: n = 184, biscuits • Study 2: n = 41, biscuits and jelly babies	The roughness of the plateware affects taste perception. Food is perceived as rougher (smoother) when sampled from a rough (smooth) plate.
Piqueras-Fiszman et al. (2011) [17]	Weight	• Study: n = 50, bowls with yoghurt	Increased bowl weight positively affects perceived density, liking, and price expectation ratings.
Piqueras-Fiszman & Spence (2012) [16]	Weight	• Study 1: n = 150, wine consumers’ beliefs • Study 2: n = 275, bottles of wine	Consumers do not consciously consider the weight of wine bottles as an indicator of price and quality. Nevertheless, the weight of the bottle correlates positively with the price of the wine.
Piqueras-Fiszman & Spence (2012) [20]	Weight	• Study: n = 45, bowls with yoghurt	Heavy (versus light) bowls increase expected satiation and perceived density of food.
Maggioni, Risso, Olivera, & Gallace (2015) [34]	Weight	• Study: n = 33, mineral water	The weight of cups affects the perception of water. Water from heavy (vs. light) cups is perceived as less pleasant and more carbonated.

Notes: n = number of subject

As the findings of prior studies indicate, the hardness of a cup (i.e., its firmness vs. flimsiness) can affect the evaluation of the beverage contained within, with inferior material quality regarding the hardness negatively affecting consumers’ product evaluations [14]. In addition, the material textural properties of the packaging in which potato chips are presented can affect the perception of crispiness and the rated tastiness [26], and potato chips in harder-to-open packages are evaluated as fresher [27]. Haptic textural cues that provide congruent vs. incongruent information affect the perceived freshness and crispness of pretzels [28]. In addition, the perceived roughness of plateware affects ratings of the mouthfeel and the taste of food [13]. Regarding the temperature cues, prior work shows that the thermal properties of beverage containers subtly affect personality evaluations [29]. For weight cues studies show that weight affects people’s judgments of volume when holding physical containers [30, 31]. The weight of a container can thus serve as a heuristic when estimating volume whereby heavier containers are perceived as being larger and thus having a greater volume [30]. In addition, previous research demonstrates that the weight of a container can influence the evaluation of the food it contains [20]. Overall, these results suggest that the haptic properties of a product’s packaging can give rise to a significant sensation transference effect on the experience and evaluation of the product itself, which gives rise to the assumption that touching the packaging of a food or beverage item may affect the perceived taste and flavor of its contents and change consumers’ product experience and response. Haptic product packaging cues may influence gustatory perception, evaluations, and, in turn, the desire for the food and beverages as well as willingness to pay.

Product cues vary with regard to their degree of diagnosticity. For example, touching a sweater provides objective information relevant to its assessment and is thus diagnostic [35]. Beyond that, research has demonstrated that non-diagnostic cues can affect consumer judgments. For example, beverage color can be more important in affecting the perceived flavor of orange juice than the actual flavor [36] and the haptic properties of a beverage container can alter perceptions of water [14]. Thus, even though irrelevant for the evaluation task, non-diagnostic product cues may alter consumer response. Empirical research on the non-diagnostic role of packaging weight for product evaluation, especially when the product packaging is disconnected from actual consumption, is scarce. Extant work suggests that the weight of an object transmits different meanings in different product categories. For consumer electronics, weight typically serves as an indicator of product quality (e.g., for remote controls [37]). However, for laptops and mobile phones, lighter devices are perceived to be of higher quality.

For containers, the weight of a bottle has been shown to positively correlate with the price of wine [16]. However, consumers do not explicitly consider bottle weight as an indicator of price and quality. In addition, the weight of tableware has been shown to affect taste evaluation. Piqueras-Fiszman et al. [16] examined the effect of the weight of a bowl on the evaluation of yoghurt. Yoghurt samples were perceived as denser and were liked more when consumed from a heavier rather than from a lighter but otherwise identical bowl.

This study adds to the latter stream of research by closing four important research gaps. First, previous studies into weight did not consider the effects for product packaging as non-diagnostic cue. Our research addresses product packaging without changing actual product weight. By providing insights into effects of weight in situations where weight and consumption are disconnected advances the extant knowledge on sensation transference, because it demonstrates that the effect can be transferred from the packaging to the product without changing intrinsic product properties.

Second, existing research has focused exclusively on either non-food or food products. No research to date focuses specifically on beverages (see [10] for a review). Foods and beverages differ in terms of their viscosity, leading to a different evolution of flavor and mouthfeel characteristics. Haptic processing differs with regard to eating versus drinking and therefore product packaging weight is processed differently. Hence, distinguishing between foods and beverages is an important step in increasing the robustness of evidence on the effects of packaging weight.

Third, previous research has mainly concentrated on packaging weight effects on product evaluations [15]. Effects on subsequent behavioral intentions have been neglected. This research contributes to the literature by studying how weight effects translate to desire for consumption. Specifically, it examines effects on the desire for food and beverage consumption caused by packaging weight.

Finally, previous investigations have so far not addressed effects of packaging weight on monetary behavioral intentions. Regarding economic validity, however, assessing impacts on willingness to pay is of utmost importance. With packaging weight often being related to costs, measuring effects on willingness to pay is an important step to validate implications.

### Hypothesis development

Flavor emerges from the interplay of taste, smell, and trigeminal (i.e., neurological) processes that relate to how sensations are transmitted to the brain, as well as possibly tactile, visual, and auditory sensations that are experienced when eating or drinking [38]. Research examining sensation transference effects for product packaging and flavor has demonstrated that packaging shape can modulate taste/flavor intensity via cross-modal correspondences serving to prime the expected taste [39, 40]. For example, participants with a high sensitivity to design rated yoghurt as tasting more intense when associated with angular rather than rounded packaging [41]. With regard to the subconscious effect of product packaging weight and ratings of flavor intensity, empirical evidence is still lacking. Studies reveal that experiencing tactile qualities activates a “haptic mindset” [42], whereby touching objects triggers the application of associated concepts. For example, studies on social behavior have revealed that holding heavy (as opposed to light) clipboards subconsciously enhances a person’s impressions of the job candidate whom they are evaluating [42].

Consistent with this line of reasoning, the present article posits that the weight of product packaging is associated with a semantically congruent weight-related metaphor activating a sensation transference effect. Analogously to distinct flavors being described in terms of shapes (e.g., ‘a sharp taste’, ‘a round wine’ [39]), intense flavors are often referred to as being heavy in everyday language such as, for example, red wines having intense flavors and aromas [43]. In addition, different sensations can share the dimension of intensity [41]. An intense sensation in one modality can subconsciously modulate the magnitude of an impression in another modality by means of sensation transference and thus increase the perceived intensity of the sensation in that modality. Recent research on fragrances lends support to this notion [44]. An intense haptic sensation caused by increased weight affects the perceived intensity of the fragrance of bath soap such that soap from heavier bottles smells more intense.

Regarding haptic perception, heaviness serves as a means to make haptic interactions more intense (“more weighty”, as the expression goes). Holding a heavy as opposed to light product may therefore alter a consumer’s expectations and suggest a more intense flavor when tasting a product. According to the subconsciousness notion of sensation transference, haptic-gustatory sensation transference effects should occur at an implicit or functionally subconscious level. Although consumers can perceive the weight of the stimulus, they will unlikely render this information meaningful. They will thus not recognize its influence on their taste perception as long as the relative weight difference compared to regular packaging weight is subtle and allows a subconscious processing. Once the weight difference of product packagings becomes obvious and is processed consciously, consumers tend to mentally correct for the effect. As such, haptically experiencing packaging heaviness at a subconscious level should affect flavor intensity. Thus:

**H1.** Heavy (vs. light) product packaging leads to a stronger (vs. weaker) perception of flavor intensity.

Prior research indicates that non-diagnostic extrinsic cues such as health claims can affect consumers’ intention to consume food (e.g., [45, 46]). This research investigates whether packaging weight as a non-diagnostic extrinsic haptic cue can alter desire for food and beverages. It is proposed that packaging weight indirectly enhances desire to consume food and beverages. The basic premise here is that heavy packaging leads to perceptions of more intense flavor. When evaluating food and beverage products, flavor intensity (up to a certain limit) serves as an indicator of quality that contributes to the perceptibility of aromas. Previous research shows that flavor intensity correlates with flavor evaluation [41, 47], that is, a valence-based gustatory appraisal of food and beverage products. Intensified flavor inspired by increased packaging weight thus leads to more favorable flavor evaluations, because particular aromas become more obvious.

Flavor is a primary factor in driving consumption behavior [48, 49]. It has been demonstrated that the liking for a food increases chewing and swallowing rates [50] and is generally at the root of excessive consumption [51]. Product names have been shown to affect the evaluation of a food item and the amount subsequently consumed [52]. Among consumers who report that they believe that healthiness and tastiness are negatively correlated, the healthy portrayal of an item negatively affects its inferred taste [53]. Among dieters, products that are perceived to be less healthy based on their names are rated as less tasty, which affects consumption behavior. In addition, the impact of external cues such as packaging and container size on consumption is mediated by taste perception and evaluation [49]. Liking a food can thus affect people’s desire to consume it. Building on these findings, haptically experiencing heaviness should increase perceived flavor intensity, which, in turn, should enhance flavor evaluation and ultimately result in a higher desire for food and beverages. However, with respect to packaged food and beverage products, haptic input plays a non-diagnostic role. Packaging weight should thus not directly affect the evaluation of flavor. Formally stated:

**H2**. Heavy (vs. light) product packaging leads to a higher (vs. lower) desire for food and beverages. This effect is serially mediated by perceived flavor intensity and flavor evaluation.

In addition to implications for the desire for consumption, this research examines effects of the weight of product packaging on consumers’ willingness to pay, that is, the maximum amount a customer is willing to spend for a product or service [54]. With increased packaging weight inspiring expectations of more intense flavor which, in turn, anchor subsequent perceptions and result in more favorable flavor evaluations, this research proposes that flavor intensity and flavor evaluation mediate the effect of packaging weight on people’s willingness to pay.

With flavor intensity being a quality indicator of food and beverages, packaging weight contributes to more favorable flavor evaluations. Monetary intentions serve as a measure reflective of consumers’ perception of a product [44]. Becker et al. established that packaging shape curvature and color saturation (seen on a computer monitor) can affect taste experiences and, in addition, more general product evaluations and price expectations.

Previous sensory marketing research on the impact of weight on monetary variables focused on expected price [15, 16]. The results of those studies that have assessed direct effects of packaging weight on expected price while not revealing significant effects, have shown positive trends associated with increases in packaging weight. Building on these initial findings, this research proposes that the effect of packaging weight on willingness to pay can be explained by integrating the mediating processes which operate when processing weight. Heavy product packaging should enhance perceived flavor intensity which, in turn, translates to more favorable flavor evaluations and subsequently results in higher willingness to pay. With packaging weight serving as a non-diagnostic extrinsic cue, there should be no direct effect of packaging weight on willingness to pay. More formally:

**H3**. Heavy (vs. light) product packaging leads to a higher (vs. lower) willingness to pay. This effect is serially mediated by perceived flavor intensity and flavor evaluation.

## Research approach

Two experimental studies tested the hypothesized effects. Study 1 used a one-factor (light vs. heavy product packaging) between-participants design and a food product (i.e., pieces of chocolate) as stimulus. Study 2 also applied a one-factor (light vs. heavy product packaging) between-participants design, but examined the effects for a beverage (i.e., soft drink). Together, the two studies provide insights into touch-taste-transference effects for both food and beverage products. Verbal informed consent was obtained from each participant prior to the experimental studies and recorded by the first author. Since all data were anonymously collected and no harming procedures were used, ethical approval was not sought for the execution of this study. The Institutional Review Board of the university approved the procedure. Participants were recruited among students of the university and they were allowed and able to opt out of the studies at any given point. None of the participants were below the age of majority.

### Study 1

#### Procedure

78 students took part in Study 1 and were randomly assigned to either a light or a heavy product packaging condition. Of the participants, 62% were female and their mean age was 23.8 years (*SD* = 2.97). The participants were instructed to take a chocolate box in their dominant hand and look at it and familiarize themselves with it for approximately 30 seconds. They then had to open the box, sample one piece of chocolate, close the box, and place it on a table. After that, the participants completed a questionnaire.

#### Stimuli and manipulation

The stimuli consisted of boxes of chocolates that were visually and haptically identical except for a subtle difference in weight. Each box contained 12 pieces of chocolate at 10g each. For the weight manipulation, a pretest was conducted based on a sample of 30 students to determine a discernible yet not outstanding difference in weight. The pretest included three boxes of chocolate: one having a regular weight, one with 30g additional weighting (+16%), and one with a 50g additional weighting (+27%). The boxes were weighted by adding lead weights to the back side of the inner layer of the product packaging. Thus, the number of pieces of chocolate per box was kept constant and the weight manipulations were not visible to the participants. In the pretest, the participants had to arrange the boxes in order of ascending weight. With the box having the intermediate weight of 30g not being correctly identified by 16.67% of the participants, the additional 50g lead weight was selected as the weight for the heavy condition as it was correctly identified by all participants. Thus the heavy box in the heavy condition had a weight of 234g (184g+50g) and the regular box in the light condition had a weight of 184g.

#### Measures

Perceived flavor intensity was captured on a 7-point Likert-type scale anchored in 1 = “not at all intense” and 7 = “very intense” [15]. Three items based on Allen et al. [55] captured flavor evaluation (i.e., “good flavor”, “great flavor”, and “flavorsome”, *α* = 0.9). These items were assessed on a 7-point Likert-type agreement scale anchored in 1 = “I strongly disagree” and 7 = “I strongly agree”. Desire for food was captured as a single-item on a five-point rating scale drawn from Small et al. [56], ranging from 1 = “eating more would make me sick” to 5 = “I really want another piece”. Willingness to pay was measured with an open-ended question where participants indicated how much they were willing to pay for one box of chocolate [14]. Of the 78 participants, eight respondents did not give an answer for willingness to pay. These participants were eliminated from the analysis regarding this outcome.

#### Results

The results of an independent *t*-test revealed a positive effect of packaging weight on perceived flavor intensity. Participants who consumed chocolate from the heavy box rated it as significantly more intense (*M* = 5.46, *SD* = 0.91) than those who consumed it from the light box (*M* = 4.74, *SD* = 1.07) (*t*(76) = 3.19, *p* < 0.01, *d* = 0.72), thus supporting H1. By contrast, there was no significant effect of packaging weight on flavor evaluation (*t*(76) = 1.06, *p >* 0.05, *d* = 0.24).

**Fig 1:**
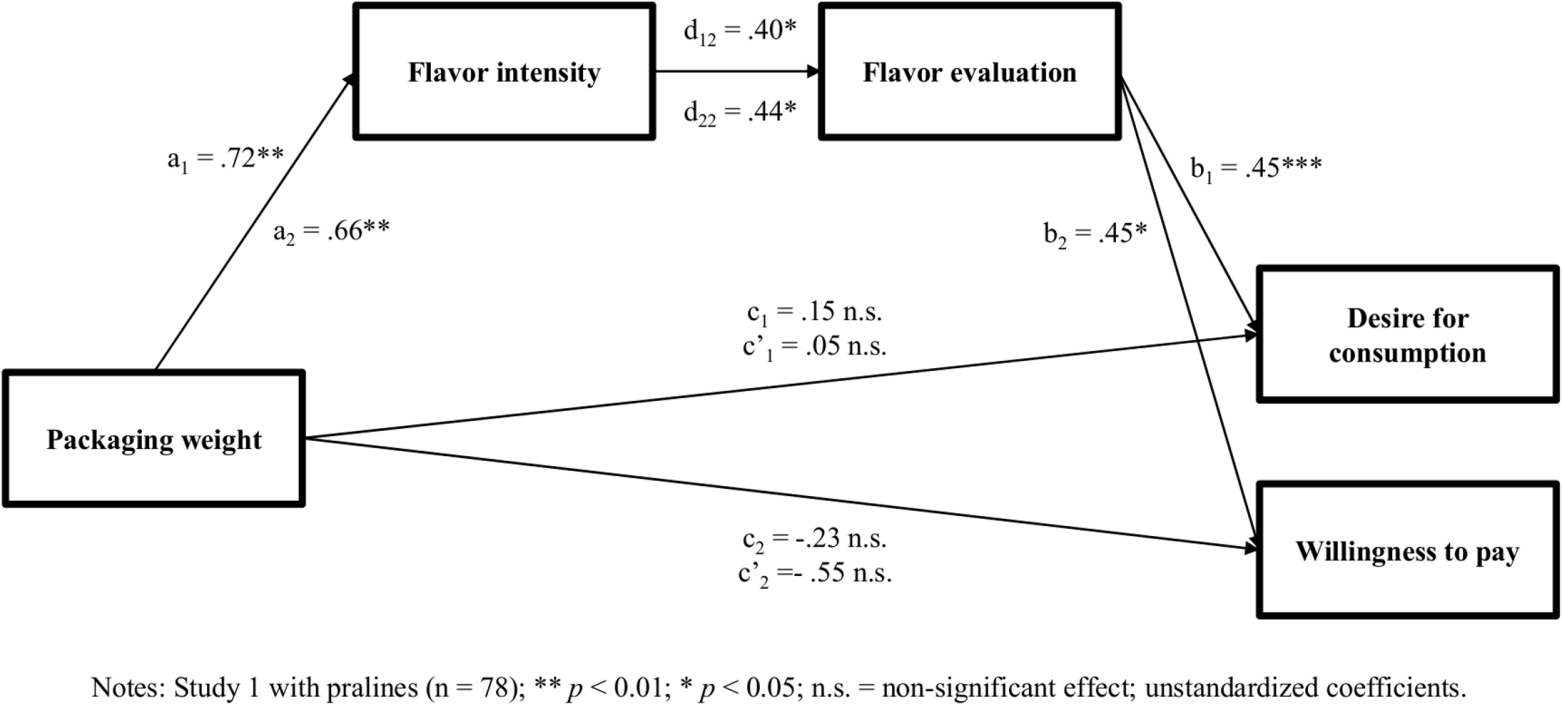
Direct and indirect effects of packaging weight on flavor intensity, flavor evaluation, desire for food, and willingness to pay.

To test H2, this study performed a serial mediation analysis by using a bootstrapping approach as recommended by Preacher and Hayes [57] and applying the PROCESS macro ([58]; model 6; 10,000 bootstrap samples; 95% confidence intervals (CI)). Mediation exists when the indirect effect of an independent variable through one or more mediators to the dependent variable is significant, that is, when the confidence interval obtained via bootstrapping does not contain the value of zero [57]. Packaging weight was the independent variable, flavor intensity and flavor evaluation (in this sequence) were the mediators, and desire for food was the dependent variable (see Fig 1). The results revealed a significant serial indirect effects of packaging weight on desire through flavor intensity and flavor evaluation with 95% CIs excluding zero (f_1_ = 0.13, *SE* = 0.07, 95% CI {0.033 to 0.306}). Specifically, the analysis revealed that packaging weight predicted flavor intensity (a_1_ = 0.72, *t*(76) = 3.19, *p <* 0.01). In addition, flavor intensity had a significant positive effect on flavor evaluation (d_12_ = 0.40, *t*(76) = 2.70, *p <* 0.05), which, in turn, positively influenced desire (b_1_ = 0.45, *t*(76) = 5.69, *p <* 0.001). Hence, H2 is supported.

Another serial mediation analysis (model 6; 10,000 bootstrap samples) tested H3. Packaging weight was the independent variable, flavor intensity and flavor evaluation (in this sequence) acted as the mediators, and willingness to pay was the dependent variable (see Fig 1). The results demonstrated that packaging weight significantly increased perceived flavor intensity (a_2_ = 0.66, *t*(68) = 2.89, *p <* 0.01) and flavor intensity had a significant positive effect on flavor evaluation (d_22_ = 0.44, *t*(68) = 2.62, *p <* 0.05), which, in turn, increased the willingness to pay (b_2_ = 0.45, *t*(68) = 2.47, *p <* 0.05). Thus, the results of the present study revealed a significant serial indirect effect of packaging weight on people’s willingness to pay through flavor intensity and flavor evaluation with 95% bootstrap CIs excluding zero (f_2_ = 0.13, *SE* = 0.08, 95% CI {0.025 to 0.396}). Therefore, H3 is supported by the data.

### Study 2

#### Procedure

74 students served as the participants in Study 2. Of the participants, 49% were female and their mean age was 22.1 years (*SD* = 2.39). The participants were randomly assigned to a light vs. heavy packaging condition and instructed to pick up a soft drink can and to familiarize themselves with it for approximately 30 seconds. Next, the participants were asked to open the can, take five small sips of the drink, and then to place the can back on the table. Finally, the participants completed the questionnaire.

#### Stimuli and manipulation

A canned soft drink (i.e., soda) served as the stimulus for Study 2. The can contained 250ml of liquid (total weight 287g). For the weight manipulation, a pretest based on a sample of 30 students was conducted to determine a discernible yet not outstanding weight difference. The pretest included three cans: one of regular weight, one with a 30g lead weight added to the outside at the bottom of the can (+10%), that is, visually invisible, and one with a 60g lead weight (+21%). Analogously to pretest 1, the participants were instructed to arrange the cans in order of weight. The results of the pretest revealed that the 60g weighting was discernible to all participants, thus this is used as the ‘heavy’ stimulus and the regular can as the ‘light’ stimulus.

#### Measures

As in Study 1, perceived flavor intensity was captured with a 7-point Likert-type scale anchored with 1 = “not at all intense” and 7 = “very intense” [15] and flavor evaluation was measured on a scale including three items (*α* = 0.92) based on Allen et al. [55]. As in Study 1, a single-item captured desire for beverages [55] and an open-ended question asked for participants’ willingness to pay. Of the 74 participants, five participants did not give an answer for willingness to pay. Hence, as in Study 1, these participants were eliminated in the analysis regarding this outcome.

#### Results

The impact of weight on flavor intensity was assessed using an independent-samples *t*-test. The results revealed a significant positive effect with participants rating the flavor of the soft drink consumed from the heavy can as significantly more intense (*M* = 4.64, *SD* = 1.60) than those who consumed the drink from the light can (*M* = 3.90, *SD* = 1.36) (*t*(72) = 2.14, *p* < 0.05, *d* = 0.50). These results therefore provide support for H1. Similar to Study 1, packaging weight did not significantly affect flavor evaluation (*t*(72) = 1.50, *p >* 0.05, *d* = 0.35).

**Fig 2:**
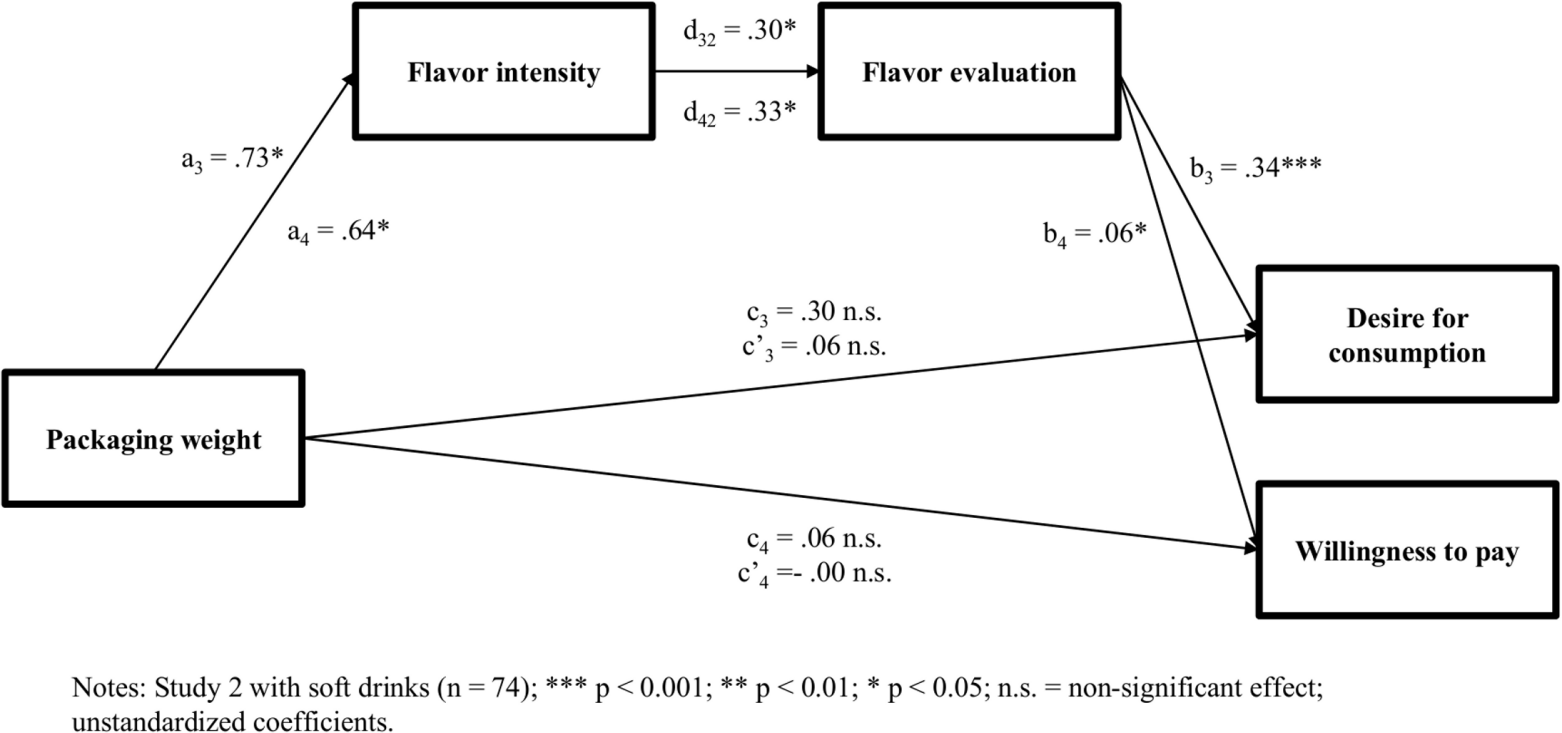
Direct and indirect effects of packaging weight on flavor intensity, flavor evaluation, desire for beverages, and willingness to pay.

To evaluate H2, a serial mediation analysis was conducted as outlined above (model 6; 10,000 bootstrap samples). Packaging weight served as the independent factor, flavor intensity and flavor evaluation as the mediators (in that sequence), and desire for beverages as the dependent variable. The results revealed a significant serial indirect effect of packaging weight on desire for consumption through flavor intensity and flavor evaluation with 95% CIs excluding zero (f_3_ = 0.08, *SE* = 0.05, 95% CI {0.008 to 0.234}). Importantly, packaging weight increased perceived flavor intensity (a_3_ = 0.73, *t*(72) = 2.14, *p* < 0.05) as proposed in H1. In addition, perceived flavor intensity had a significant positive effect on overall flavor evaluation (d_32_ = 0.30, *t*(72) = 2.26, *p* < 0.05), which, in turn, had a significant positive effect on the desire for beverages (b_3_ = 0.34, *t*(72) = 6.48, *p* < 0.001). As depicted in Fig 2, these findings demonstrate the intervening roles of flavor intensity and flavor evaluation in the relationship between packaging weight and desire for beverages.

To evaluate H3, this study performed another serial mediation analysis (model 6; 10,000 bootstrap samples). Packaging weight was specified as the independent factor, flavor intensity and flavor evaluation as the mediators (in that sequence), and willingness to pay as the dependent variable. The results demonstrated that packaging weight significantly increased perceived flavor intensity (a_4_ = 0.64, *t*(67) = 1.76, *p* < 0.05). Flavor intensity, in turn, was found to significantly enhance overall flavor evaluation (d_42_ = 0.33, *t*(67) = 2.49, *p <* 0.05), which then led to a greater willingness to pay (b_4_ = 0.06, *t*(67) = 1.69, *p <* 0.05). Thus, the results revealed a significant serial indirect effect of packaging weight on willingness to pay through flavor intensity and flavor evaluation with 95% CIs excluding zero (f_4_ = 0.01, *SE* = 0.01, 95% CI {0.0002 to 0.0506}). In accordance with H3, participants who consumed drinks from heavy (vs. light) product packaging experienced greater flavor intensity, evaluated the flavor more favorably, and reported a higher willingness to pay (see Fig 2).

## Discussion

Multisensory product design and consumer haptic perception studies show that the perception of product packaging influences how consumers evaluate products (e.g., [3,4]). However, existing research has so far paid more attention to the texture and hardness of the packaging (e.g., [14, 25] than to its weight. The weight of product packaging has not been studied before. Previous research has focused on the weight of tableware (e.g., [15, 20]). However, tableware is not directly related to the product consumed. This research examined product packaging as an inherent part of the global product offering at the moment of purchase and consumption. Thus, this article is the first to demonstrate an effect of packaging weight on flavor perception, flavor evaluation, as well as subsequent desire for food and beverages and willingness to pay. Building on these findings, the present study provides several contributions to the related stream of literature.

First, the present research examines the role of packaging weight for flavor perceptions (i.e., flavor intensity and flavor evaluation). In line with hypothesis H1, the results show that packaging weight as a positively valenced non-diagnostic cue is positively associated with perceptions of flavor intensity, that is, heavier weight triggers more intense flavor ratings.

Second, this research investigates effects of packaging weight on desire for food and beverages and willingness to pay. In line with hypotheses H2 and H3, it finds that weight as a non-diagnostic haptic packaging cue has a positive indirect effect on consumer response. To the best of the authors’ knowledge, this research is also the first to study this relationship empirically. The results support the account that the impact of packaging weight on desire for consumption and willingness to pay is mediated by consumers’ perceptions of flavor intensity and flavor evaluation. With flavor intensity generally representing an important indicator of quality when evaluating food and beverages, higher experienced intensity positively affects overall flavor evaluation and subsequent consumer response.

Taken together, these findings provide empirical evidence that supports this study’s conceptual framework that is grounded in extant sensation transference research. Moreover, it extends previous findings by demonstrating the interplay between haptic perception and behavioral intentions. Thus, this research explains the mediating processes that operate when consumers process product weight as well as the related haptic-gustatory sensation transference effects.

These findings also provide arguments to managers discussing the economic effects of increases in packaging weight. First, they show that higher packaging weight induces a higher desire for food and beverages and might thus increase sales volume. Second, they demonstrate that while heavier packaging may lead to cost increases, these additional expenses may be balanced or possibly overcompensated by consumers through an increased willingness to pay.

Third, this article is the first to corroborate sensation transference effects for both food and beverages. Previous investigations have focused solely on food and thus failed to account for different properties of solids and liquid foodstuffs. The food and beverage products selected here include solids (i.e., pieces of chocolate) and liquids (i.e., soft drinks). These products differ from in-between forms such as yoghurt in their intrinsic characteristics. In addition, the products used in the studies were not changed, but only differed with regard to their packaging. Moreover, they can be directly consumed from the packaging. In previous investigations, a spoon had to be used to transfer yoghurt from a bowl to the mouth (e.g., [15]). The weight of the cutlery a food is consumed with can affect food liking and willingness to pay [59]. Thus, the interaction with the cutlery may have sidetracked potential effects. This research contributes to the literature by focusing on foods and beverages that consumers can consume directly from the containers (i.e., the product packaging) without any use of cutlery, thus demonstrating the sensation transference effect from packaging to consumption in situations where weight is disconnected from consumption. Fourth, one might object that increasing packaging weight doesn’t only have economic consequences in the form of cost increases. It also may produce undesirable ecological consequences. Firms operating in highly competitive industries, such as the food and beverage sector, are increasingly inclined to use haptically-inferior packaging in order to generate cost savings and to conform to international packaging regulations [60]. Managers in this context frequently disregard the consumption experiences of consumers. The findings of this article show, however, that even moderate changes of the weight of product packaging can alter how consumers experience products and respond to them. In contrast to previous studies that have examined the effect of considerable differences in tableware weight (e.g., [15], weight increase: 80%), this research demonstrates that a much more modest modification of the weight (specifically a weight increase of 27% in Study 1 and 21% in Study 2) has a positive effect on perceived flavor intensity, flavor evaluation, and subsequent desire for food and beverages and willingness to pay. This knowledge may help packaging designers to create more ‘flavorful’ packaging for food and beverage products while limiting the increase in packaging weight. Product-specific studies would need to be conducted in order to identify the specific thresholds above which significant positive effects on consumer perceptions and behavior can be observed in different product categories. It may well be that much smaller weight increases are sufficient than the approximately 20% applied in this research and the 80% used in other research. Note that an increase in packaging weight does not necessarily imply a more negative ecological impact of the packaging. For example, if manufacturers move to heavier packaging (e.g., glass instead of plastic) this may be the basis for the introduction of a circular system in which packaging is returned and re-used rather than thrown away. Hence, if accompanied by more radical transformations of the underlying packaging life-cycle logic, increases in packaging weight may even lead to a reduced overall ecological footprint.

Fifth, the results obtained in this research also have implications for food or beverages supporting a healthy lifestyle. For example, with regard to products that might be perceived as less intense in flavor (e.g., due to a reduction in sugar, fat, or calories), increasing the weight of the packaging might compensate for this negative perception and have a positive effect on perceived flavor intensity. For example, with fruit and vegetables often having little if any packaging weight, an increase of packaging weight could lead to both increased sales and consumption within that product category. Packaging weight might thus help counter the ‘unhealthy = tasty intuition’ [53] according to which the less healthy an item is portrayed to be, the better is its inferred taste, by positively contributing to flavor perception and evaluation. With marketers being easily able to change haptic properties, packaging weight can thus be thought of as offering a ‘healthy’ way to improve flavor evaluation and perhaps foster a diet that is slightly more balanced. Using such techniques might serve as a means to help improve the perception and evaluation of healthy products and, thus, counter increasing obesity rates [19, 61].

Sixth, beyond inspiring more intense flavor perceptions and improving flavor evaluation, increased packaging weight also leads to a higher desire for food and beverages. Consumers will therefore not only enjoy those foods and beverages more, but potentially consume more. With regard to managerial implications, this finding might result in higher sales volumes triggered by increased packaging weight. With regard to consumer implications for healthy foods and beverages, this finding might contribute to a healthier lifestyle by leading consumers to increase their consumption of healthy products. Regarding unhealthy products, responsible marketers and individual consumers can take steps to control consumption volume. For example, marketers can guide consumers’ consumption intentions by lowering the packaging weight. The findings of this study suggest that with packaging weight decreasing consumers are likely not only to perceive flavors as less intense and less favorable, but eventually show a lower desire to consume those food and beverage products. Beyond that, consumers can make changes to their personal consumption habits to help reduce their consumption intentions. By avoiding direct consumption out of the packaging (e.g., by drinking out of a light cup rather than directly from a can), they can perhaps try to impede the sensation transference effect of packaging weight.

Seventh, the findings show that packaging weight positively affects consumers’ willingness to pay. This effect is of particular interest with regard to sales revenue and potential profits. Packaging weight is often associated with raw material, manufacturing and logistics costs. In many industries, managers increasingly focus on cutting down costs in order to preserve competitiveness. However, higher packaging weight resulting in higher willingness to pay, increased packaging weight may actually an alternative to cost cutting programs or at least provide an additional lever in the quest for competitive advantage.

It is worth remembering that the focus of the studies reported here was on hedonic products. The findings might thus not necessarily be applicable to all types of food and beverages (e.g., utilitarian foods). Additional research is undoubtedly needed in order to study the effect of the weight of product packaging on flavor evaluation for utilitarian products such as, for example, crisp bread. With the results showing that packaging weight can increase perceived intensity for sweet products, it would also be interesting for future research to address the question of whether increasing the weight of the packaging can increase perceived sweetness. Increasing sweetness perception by external non-diagnostic cues might enable producers to cut down actual sugar content even further than many of them have already started to do.

With regard to future research advancing theory, it would be interesting to assess whether haptic-gustatory sensation transference effects also operate on a more conscious level. Based on the theoretical foundation, the effect is likely to disappear when weight differences become too obvious to operate on an unconscious level of processing, as consumers will then be able to mentally correct for it [18]. Similarly, making participants aware of the packaging weight (i.e., by applying a within-participants design or by directly asking for perceived weight), effects may vanish, as participants would detect the weight increase and thus no longer associate higher packaging weight with more intense tastes subconsciously. Moreover, further research into the concept of priming heaviness is needed. For example, is there a difference between priming heaviness by a haptic perception (i.e., holding something) or by a mental activation (i.e., using words) in terms of embodied cognition when assessing subsequent gustatory evaluations? Research into that area would further elucidate the operating mechanisms of haptic-gustatory sensation transference effects.

Regarding future research with managerial implications, additional work is required to better understand the interplay between the weight of product packaging and consumer response. First, an important open question in this regard is whether there is a habituation effect of packaging weight caused by mere exposure. This research assessed a positive influence of packaging weight using a ‘single shot’ experimental procedure. It is, however, not yet clear whether the effects in desire for consumption and willingness to pay would still occur after several consumption episodes. Second, investigating real behavior would contribute to a better understanding of the observed haptic-gustatory sensation transference effects. This research studied packaging weight effects on desire for food and beverages and willingness to pay. To corroborate these findings, an interesting extension of this work would thus be to assess actual consumption and price behavior in a realistic setting. Third, studying packaging weight effects for different flavor dimensions and product categories would likely shed more light into underlying mechanisms. Whereas for pleasant flavors, increased weight has demonstrated to exert positive effects on flavor and in turn flavor experience, the opposite might hold true for unpleasant flavors or products, such as medicine or coffee being perceived as too bitter, vinegar too sour, or mustard too hot. Fourth, a further line of experimental research could involve examining whether packaging weight still influences flavor perception when consumers pour the contents of heavier product packaging into another receptacle (e.g., a glass) before tasting. If, for example, a consumer pours a drink from a heavier can (or bottle) into a glass, the weight is experienced at both spatial and temporal distance from the actual consumption experience. Therefore, the influence of the product packaging would be expected to be smaller.

Additionally, examining whether the observed effects come directly from perceptions of increased packaging weight or from higher perceived total product weight would be a fruitful avenue for further research. In order to disentangle packaging from product weight, it would thus be important to assess effects by specifically eliminating a possible inference to product weight. For example by studying consumer response to empty chocolate boxes or soda cans of different weight, effects could be more clearly reducible to packaging weight. Previous findings give rise to the indication that packaging weight is attributed to total product weight. A study investigating effects of yoghurt consumed from bowls found that despite a change of bowl weight (instead of product weight) consumers perceive yoghurt consumed out of the heavier bowl as heavier and thus denser [15].

Moreover, further research is needed to investigate whether packaging weight enhances flavor perception and consumer response when the consumer is faced with the increased weight when carrying shopping items before consuming the food or beverage. Examples from the beverage industry show, for example, that Sapporo, a Japanese brewery, successfully introduced a heavier steel can to the market, and that sales increased dramatically afterwards [62].

With packaging decisions and resulting implications becoming of increasing importance to managers and spawning much recent consumer research, this article adds to this growing body of research by bringing insight to the effect of product packaging weight on consumer response.
